# Supplementary material for: Stringent sustainability regulations for global supply chains are supported across middle-income democracies
Source: Nat Commun. 2024 Feb 5;15:1049. doi: 10.1038/s41467-024-45399-5 (PMC10844325; doi:10.1038/s41467-024-45399-5)
Supplement: Supplementary file 1 — Supplementary Information [file 41467_2024_45399_MOESM1_ESM.pdf]

Supplementary Information

Stringent sustainability regulations for global supply chains are supported across middle-income democracies

E. Keith Smith, Dennis Kolcava and Thomas Bernauer

Notes - Expectations Elaboration and Motivation

S1 Macro- and Individual-Level Economic Conditions

The 'Environmental Kuznets Curve' (EKC) is commonly used to illustrate the relationship between economic development and pollution levels [1], and has more recently been extended to explore 'generalised' negative sustainability externalities (e.g. basic health and workplace standards) [2]. The EKC's macro-level mechanisms imply that countries tend to follow an initial path of intensive economic expansion ('scale' effect) at the expense of domestic environmental and social public goods. However, with progressive macroeconomic development, countries are becoming increasingly reliant on the services sector ('composition effect') and cleaner (safer) production for the generation of value ('technology' effect)[3, 4]. Hence, at an intermediate level of development, the composition, and technology effects overcompensate for the scale effect, instigating a decline in externalities.

Various extensions link the main EKC macro-level dynamics to micro-level consequences. First, at higher levels of affluence, environmental and social public goods transition from being perceived as 'necessity' to 'luxury' goods by citizens [5, 6] – implying increased support for public policy to provide these goods. Such development arguments mirror post-materialist perspectives [7].

Second, the comparative advantage in pollution- or labour-intensive production within lower- and middle-income countries likely plays a role in citizens' preference formation as well – a notable factor in the context of global supply chain regulation. Increases in trade and foreign direct investment (FDI) represent substantial economic value-added [8, 9]. Additionally, from the perspective of standard trade preference formation models, global supply chain policy acceptability is likely to be conditioned by individuals' level of education and training, which is a crucial dimension of individuals' factor endowment [10], as the level of education likely determines individual dependence on labour- or pollution-intensive economic activity. To the extent to which regulating global supply chains reduces non-OECD countries' comparative advantage in pollution- or labour-intensive production, individuals holding lower levels of education and training should be less supportive of stringent supply chain policy.

As a result, citizens in non-OECD countries are likely to face disincentives – be it for individual economic reasons or rather due to concerns for broader societal economic outcomes at large [11] – to support stringent sustainability regulation for multinationals and their subsidiaries along global supply chains. This baseline approach suggest that new global supply chains rules may rather be viewed as a threat to economic activity in economically developing countries.

> [Expectation 1]: Public support for supply chain sustainability regulations will be misaligned between high-, and middle-income countries – public support being lower in middle-income than in high-income countries.

S2 Policy Benefit and Cost Perceptions

In democratic states, policymakers are driven towards developing regulatory frameworks that optimise political support (and thus minimise resistance). Recent research has explored how the acceptability of sustainability regulation of global supply chains varies across forms of policy instruments [12, 2]. Broadly, the acceptability of policy instruments is assumed to increase in relation to perceived benefits, and diminish with heightened measures of coercion and increased visibility of perceived costs [13, 14].

However, policy instruments that have the highest levels of acceptability are often those with the lowest expected impacts and cost-efficiencies [15]. In contrast, instruments that internalise externalities more efficiently are often those with the lowest acceptability [16]. Accordingly, a potential dilemma arises between the acceptability and effectiveness of sustainability regulation, where, in the worst case, the most effective interventions are political non-starters and the most acceptable interventions are ineffective ('ineffectiveness by design') [17].

> [Expectation 2]: Citizens will associate larger cost and benefit perceptions with more stringency policy packages – high stringency designs will have larger perceived benefits (e.g., in terms of improving local production conditions), while at the same time they will be associated with greater perceived costs (e.g., to firms and their employees, and consumers).

S3 Individual Characteristics

Citizens of democracies have commonly been presumed to prioritize policies that increase economic growth over those safeguarding environmental (e.g. biodiversity, clean air) and social public goods (e.g. safe working conditions) [18, 19]. However, citizens with lower levels of individual economic conditions (such as income) may be less likely to support new measures over concerns regarding potential increased consumer costs or job losses. However, recent research suggests that support for environmentally-related policies in non-OECD democracies is not a mere function of individual economic conditions (such as income), and rather perceptions of environmental quality and environmental attitudes matter a great deal [20, 21]. These debates mirror sociological debates regarding the heterogeneous effect of economic development [22, 23] – especially, whether affluence increases environmental concern [24] or whether non-economic factors [25] such as exposure to environmental problems shape attitudes and resulting policy preferences. Accordingly,

we explore how policy preferences vary by environmental attitudes, educational attainment, and household income.

> [Expectation 3]: Individual-level environmental concerns and perceived impacts will be positively associated with support for more stringent policies.

S4 Robustness of Supply Chain Policy Preferences

Given the increasing political salience of regulating business conduct within non-OECD contexts, we expect that respondents will have formulated supply chain policy preferences before participating in the survey. As supply chain policy preferences are relatively well-formulated, recent empirical evidence suggests a limited potential for manipulation by information provisioning [12]. Accordingly, we expect that preferences will be largely robust against such manipulation, and, therefore, we implement several informational treatments as a robustness check.

> [Expectation 4]: Supply chain policy preferences will be robust against information on potential costs and benefits associated with these measures.

Tables

Table S1: Conjoint Introduction Wordings

Conjoint Intro Step – Part 1	OECD (United States Example)	Non OECD (India Example)
Intro Step 1	Products sold in the United States are often made partially or entirely in another country. Think for example of coffee, computers, clothes, cars, gold, chemicals, or steel. These products are manufactured and sold through international supply chains that may involve multiple companies from the United States and from other countries abroad.	Products made in India are often sold in Europe or North America. In 2019, India exported $330 billion dollars' worth of products overseas. Think for example of refined petroleum, diamonds and jewelry, chemicals, clothes, and electronics.
Intro Step 2	Each country has their own distinct standards for labour conditions and environmental protections. Hence, imported products that we buy locally in the United States may be produced abroad under certain working (i.e. wages, working hours, safety rules) and environmental conditions (i.e. clean air and water quality) that are different than the United States' standards.	Each country has their own standards for working conditions and environmental protection. Some Indian-made products meet or exceed the standards for working conditions and environmental protection of the countries where these products are eventually sold (for example in Europe or North America). Other Indian-made products, however, do not meet those standards.

Intro Step 3	No text	Many governments in Europe and North America are currently adopting new laws for companies that import goods into their country. These laws would require companies to disclose more information about the working and environmental conditions under which imported products are made overseas in countries like India. Soon companies will only be allowed to sell imported products in Europe and North America that meet these increased standards for working conditions and environmental protection.
Intro Step 4	Politicians and societies around the world are debating whether governments should adopt and enforce new laws requiring companies that sell imported products to disclose more information about the local environmental and working conditions in their international supply chains. They are also debating which companies such new laws should apply to and how strict these new laws should be. We would like to know what you think. On the following pages, we will show you some of the main discussion points in these debates on regulating international supply chains.	The Indian government could adopt similar laws to guarantee that all products made in India meet the increased European and North American standards. We would like to know what you think. On the following pages, we will show you some of the possibilities for new laws requiring companies in India to disclose more information about the local working and environmental conditions where their products are made in India. These laws differ by the size of the company they could apply to, the amount of information the company is required to provide, and what governments can do if companies do not comply with these new laws.

Table S2: Overview of Policy Design Components in the Conjoint Experiment

**Components**	**Values**
Scope – A new law could apply to:	• Very large companies (25,000 employees or more)<br>• Large and very large companies (2,500 employees or more)<br>• Medium sized, large and very large companies (250 employees or more)<br>• All companies with 25 employees or more
Transparency – A new law could make companies' reporting:	• Slightly stricter – no government rules on required content (companies can freely choose what they report), annual confidential report to government<br>• Somewhat stricter – some general government rules on required content (companies can partially choose what they report), annual confidential report to government<br>• Much stricter – detailed government rules on required content (companies must report according to a specific set of questions), annual public report (online)
Enforcement – If a company withholds or presents false information, a new law could:	• Not change the status quo and not allow for government action against the company. The company can only remind the company of its responsibility.<br>• Allow for some government action – putting the company on a public list of companies that provide unreliable information and imposing a moderate financial penalty<br>• Allow for strong government action – putting the company on a public list of companies that provide unreliable information, imposing a severe financial penalty, stop buying government supplies from that company, press legal charges against the company management.

Table S3: **Expected Policy Benefits and Costs Statements**

**Dimension**	**Working Conditions**	**Environmental Conditions**
Better Information	This new law would guarantee companies provide accurate information to consumers about the working conditions under which these products were made in [COUNTRY].	This new law would guarantee companies provide accurate information to consumers about the environmental conditions under which these products were made in [COUNTRY].
Better Production	This new law will help create more fair local working conditions in [COUNTRY].	This new law will help protect the local environment in [COUNTRY].
Job Creation	This new law would make it easier for products made in [COUNTRY] to be exported abroad and would create jobs in [COUNTRY].	
Firm Costs	This new law would increase administrative burdens, would deter companies from doing business, and put jobs in [COUNTRY] at risk.	
Consumer Costs	This new law would hurt consumers in [COUNTRY] by making products more expensive.	
National Sovereignty	This new law would force businesses in [COUNTRY] to adopt new rules dictated by countries in Europe and North America.	

Table S4: Wording of the vignette treatments (example of India)

**Treatment**	**Wording**
Improved conditions	Supporters of these new laws say that they will improve working conditions and help protect the environment in India.
Increased trade and jobs	Supporters of these new laws say that they will help increase the amount of products exported from India to Europe and North America, creating additional jobs in India.
Consumer costs and job loss	Opponents of these new laws say that they will increase consumer prices in India, reduce profits for Indian companies, and put jobs in India at risk.
Threats to sovereignty	Opponents of these new laws say that they will limit India's sovereignty, forcing companies to meet new rules dictated by foreign countries.

Table S5: Distribution of Sample by Gender interlocked with Age and Educational attainment for Brazil, Indonesia and India. Displaying population distribution compared with survey sample.

	BR		ID		IN	
Pop.	Pop. Sample	Sample Pop.	Pop. Sample	Sample Pop.	Pop. Sample	Sample
Male						
18-24	0.08	0.08	0.09	0.09	0.11	0.11
25-34	0.12	0.12	0.14	0.14	0.13	0.13
35-44	0.10	0.10	0.11	0.12	0.11	0.11
45-54	0.08	0.08	0.08	0.08	0.07	0.07
55-64	0.05	0.05	0.05	0.05	0.05	0.05
65+	0.05	0.05	0.04	0.04	0.04	0.04
Female						
18-24	0.09	0.09	0.09	0.09	0.10	0.10
25-34	0.13	0.13	0.13	0.13	0.13	0.13
35-44	0.10	0.10	0.11	0.11	0.10	0.10
45-54	0.08	0.08	0.08	0.08	0.07	0.07
55-64	0.06	0.06	0.05	0.05	0.05	0.05
65+	0.05	0.05	0.04	0.04	0.04	0.04
Education						
Low	0.51	0.37	0.64	0.62	0.71	0.68
Moderate	0.34	0.40	0.26	0.27	0.18	0.19
High	0.15	0.23	0.10	0.11	0.11	0.14

Table S6: **Attention Checks**.

	**Attention Check**	**Correct Response**
50% Median Time	7m 08s	>7m 08s
Attention Check 1	To show that you are reading the questions carefully, please indicate below how many wheels a bicycle has	2
Attention Check 2	”Which age group do you belong to?” and ”To show that you are reading the questions carefully, please enter your year of birth below.”	Stated age fits within age category

Table S7: Distribution of Sample by Gender interlocked with Age and Educational attainment for Belgium, Canada, Switzerland, Germany, Spain and France. Displaying population distribution compared with survey sample.

	BE		CA		CH		DE		ES		FR	
	Pop.	Sample	Pop.	Sample	Pop.	Sample	Pop.	Sample	Pop.	Sample	Pop.	Sample
Male												
18-24	0.06	0.06	0.06	0.06	0.06	0.06	0.05	0.05	0.05	0.05	0.06	0.06
25-34	0.09	0.09	0.09	0.09	0.09	0.09	0.08	0.08	0.10	0.10	0.09	0.09
35-44	0.09	0.09	0.09	0.09	0.10	0.10	0.09	0.09	0.12	0.12	0.10	0.10
45-54	0.10	0.10	0.10	0.10	0.11	0.11	0.12	0.12	0.10	0.10	0.10	0.10
55-64	0.09	0.09	0.09	0.09	0.08	0.08	0.09	0.09	0.07	0.07	0.09	0.09
65+	0.07	0.07	0.06	0.06	0.07	0.06	0.07	0.07	0.06	0.06	0.06	0.06
Female												
18-24	0.06	0.06	0.06	0.06	0.06	0.06	0.05	0.05	0.05	0.05	0.06	0.06
25-34	0.08	0.08	0.09	0.09	0.09	0.09	0.08	0.08	0.09	0.09	0.09	0.09
35-44	0.09	0.09	0.09	0.09	0.10	0.10	0.09	0.09	0.12	0.12	0.09	0.09
45-54	0.10	0.10	0.10	0.10	0.11	0.11	0.11	0.11	0.10	0.10	0.10	0.10
55-64	0.09	0.09	0.09	0.09	0.08	0.08	0.09	0.09	0.08	0.08	0.09	0.09
65+	0.07	0.07	0.07	0.07	0.06	0.06	0.08	0.08	0.07	0.07	0.07	0.07
Education												
Low	0.25	0.21	0.09	0.06	0.12	0.08	0.13	0.13	0.41	0.30	0.22	0.22
Moderate	0.37	0.40	0.35	0.38	0.46	0.50	0.58	0.58	0.23	0.32	0.43	0.43
High	0.37	0.40	0.35	0.38	0.46	0.50	0.58	0.58	0.23	0.32	0.43	0.43

Table S8: Distribution of Sample by Gender interlocked with Age and Educational attainment for Italy, Japan, South Korea, Netherlands, United Kingdom and United States. Displaying population distribution compared with survey sample.

	IT		JA		KO		NL		UK		US	
	Pop.	Sample	Pop.	Sample	Pop.	Sample	Pop.	Sample	Pop.	Sample	Pop.	Sample
Male												
18-24	0.05	0.05	0.05	0.05	0.07	0.07	0.06	0.06	0.07	0.07	0.07	0.07
25-34	0.08	0.08	0.08	0.08	0.10	0.10	0.08	0.08	0.10	0.09	0.10	0.10
35-44	0.11	0.11	0.10	0.10	0.11	0.11	0.09	0.09	0.09	0.09	0.09	0.09
45-54	0.10	0.10	0.09	0.09	0.11	0.11	0.10	0.10	0.10	0.10	0.10	0.10
55-64	0.08	0.08	0.10	0.10	0.08	0.08	0.09	0.09	0.08	0.08	0.08	0.08
65+	0.07	0.07	0.09	0.09	0.05	0.05	0.07	0.07	0.07	0.07	0.05	0.05
Female												
18-24	0.05	0.05	0.05	0.05	0.06	0.06	0.06	0.06	0.06	0.06	0.07	0.07
25-34	0.08	0.08	0.08	0.08	0.09	0.09	0.08	0.08	0.09	0.09	0.10	0.10
35-44	0.11	0.11	0.10	0.10	0.11	0.11	0.09	0.09	0.09	0.09	0.09	0.09
45-54	0.11	0.11	0.09	0.09	0.11	0.11	0.10	0.10	0.10	0.10	0.10	0.10
55-64	0.09	0.09	0.10	0.10	0.08	0.08	0.09	0.09	0.08	0.08	0.09	0.09
65+	0.08	0.08	0.10	0.10	0.06	0.06	0.07	0.07	0.07	0.07	0.06	0.06
Education												
Low	0.40	0.40	0.01	0.01	0.13	0.05	0.23	0.07	0.19	0.17	0.10	0.10
Moderate	0.42	0.42	0.49	0.49	0.40	0.48	0.41	0.53	0.35	0.32	0.44	0.44
High	0.42	0.42	0.49	0.49	0.40	0.48	0.41	0.53	0.35	0.32	0.44	0.44

Table S9: Items and polychoric factor loading of Environmental Concern

**Item**	**Factor Loadings**			**Uniqueness**
	(1)	(2)	(3)	
(1) It is just too difficult for someone like me to do much about the environment.	0.79	0.17	-0.09	0.33
(2)There are more important things to do in life than protect the environment.	0.80	-0.26	-0.07	0.28
(3) There is no point in doing what I can for the environment unless others do the same.	0.73	0.03	-0.01	0.47
(4) Many of the claims about environmental threats are exaggerated.	0.74	-0.20	0.12	0.40
(5) I find it hard to know whether the way I live is helpful or harmful to the environment.	0.68	0.29	0.08	0.45
Eigenvalue	2.82	0.22	0.04	.
Difference	2.60	0.18	0.04	.
Proportion	0.92	0.07	0.01	.
Cumulative	0.92	0.99	1.00	.

Figures

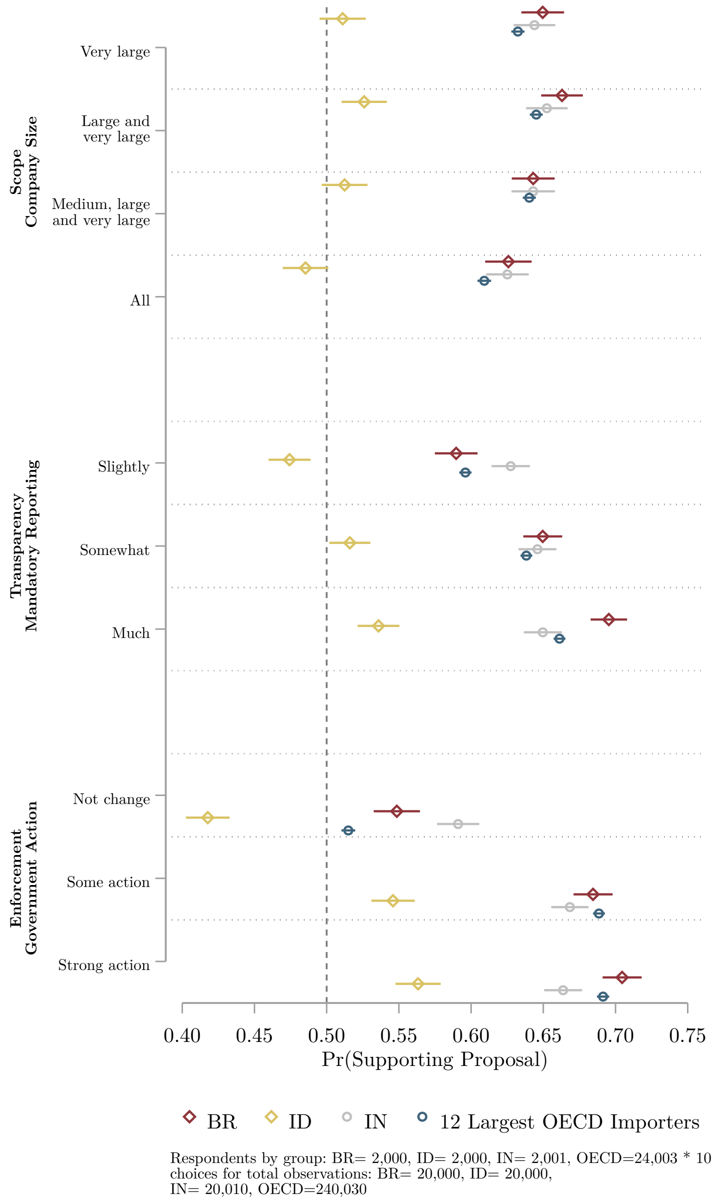

Figure S1: **Support for global supply chain policy instruments in BR, ID, IN, and the 12 largest OECD countries (by imports). Marginal means of predicted support for policy instruments are plotted for each region with 95% confidence intervals.**

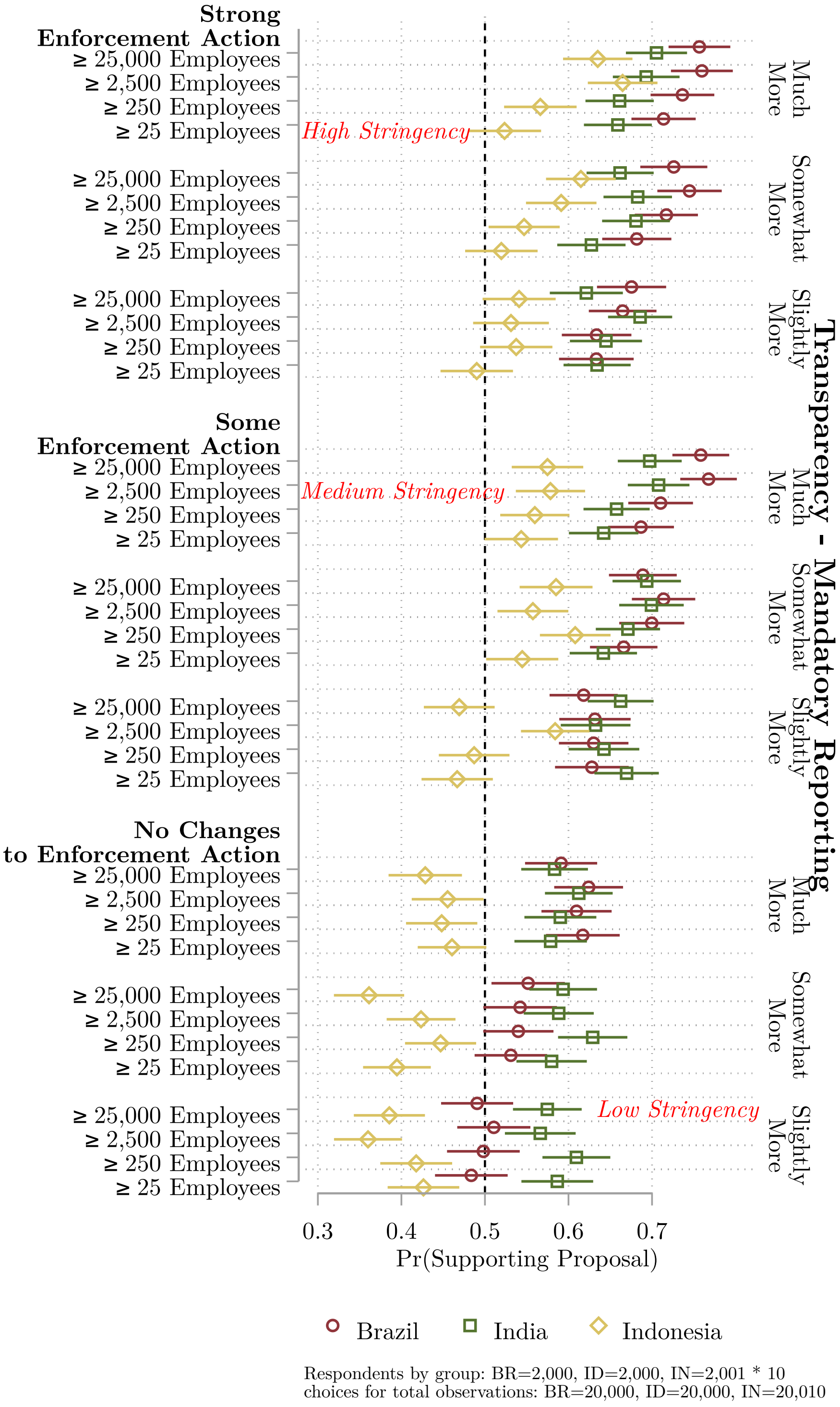

Figure S2: **Support for global supply chain policy**. Marginal means (predicted probabilities) are calculated from the conjoint choice experiment of policy packages for full factorial, interactive product terms of all policy instruments, with 95% confidence intervals (respondent-level clustered standard errors). Individual choice based on support for a policy proposal (A or B) is the dependent variable. 50% probability of supporting a proposal with the given characteristics is plotted in black. Policy stringency packages utilised for analyses in main text are highlighted in red text.

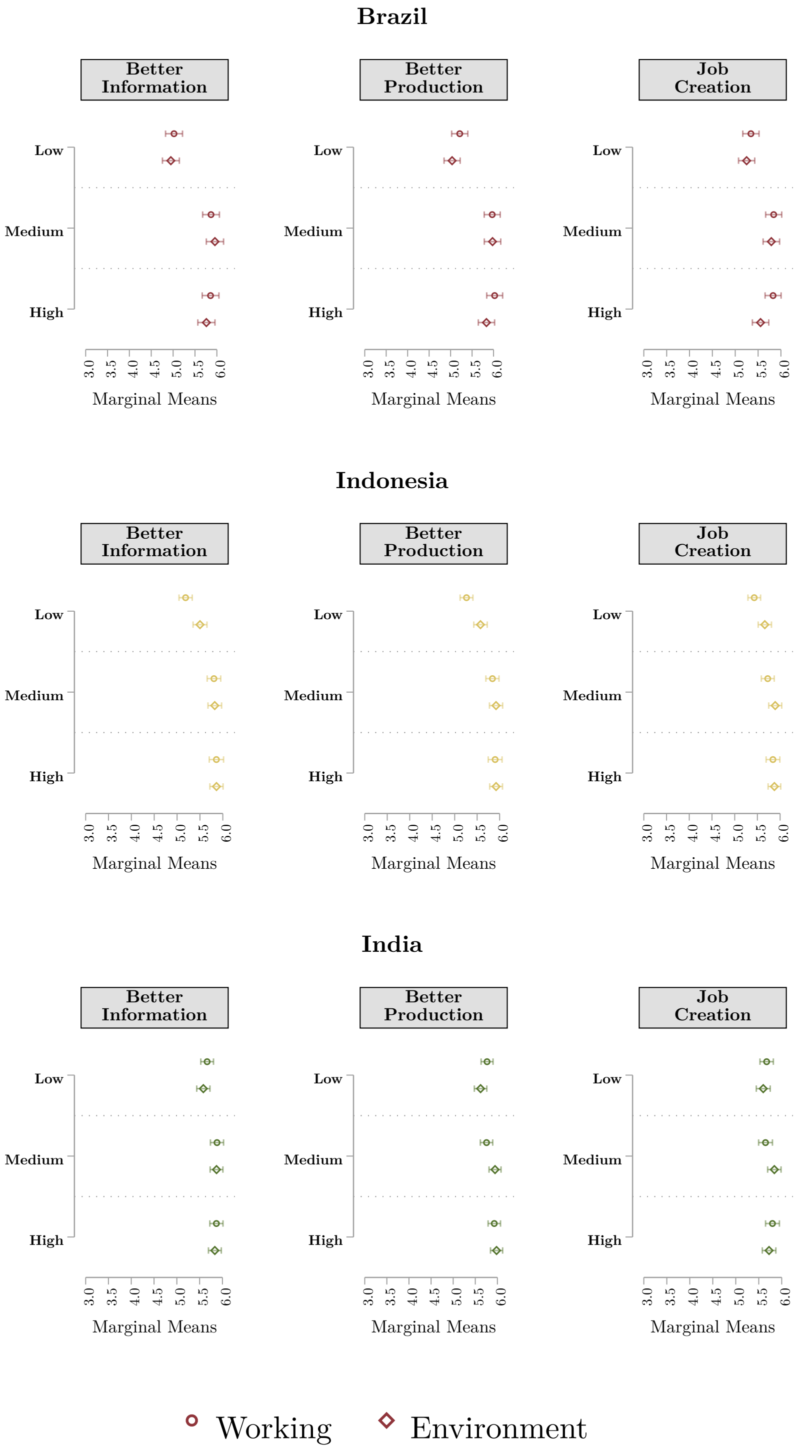

Figure S3: **Perceptions of benefits associated with global supply chain policies by regulatory stringency by ”Working” and ”Environmental” conditions treatments in BR, ID, and IN. Marginal means of perceptions are plotted by experimentally assigned regulatory stringency packages, with 95% confidence intervals plotted in dashed lines.**

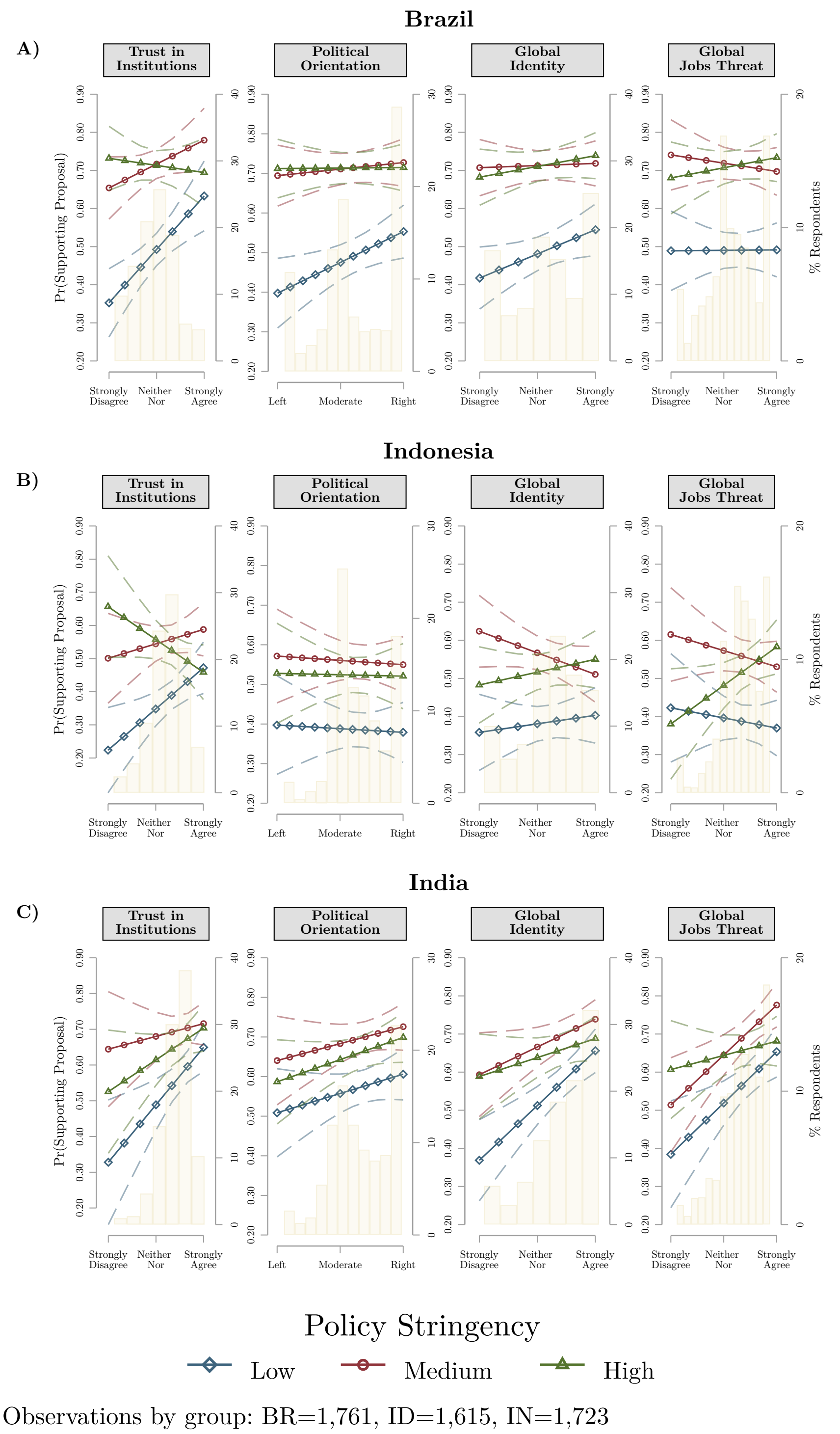

Figure S4: Support for global supply chain policies in BR, ID, IN, and the 12 largest OECD countries (by imports) by subgroup characteristics. Marginal means of predicted support (left y-axis) for policies by regulatory stringency are plotted for each region, with 95% confidence intervals in dashes by different subgroup characteristics. The distributions of subgroup characteristics by percentage of respondents (right y-axis) are plotted in yellow for each region.

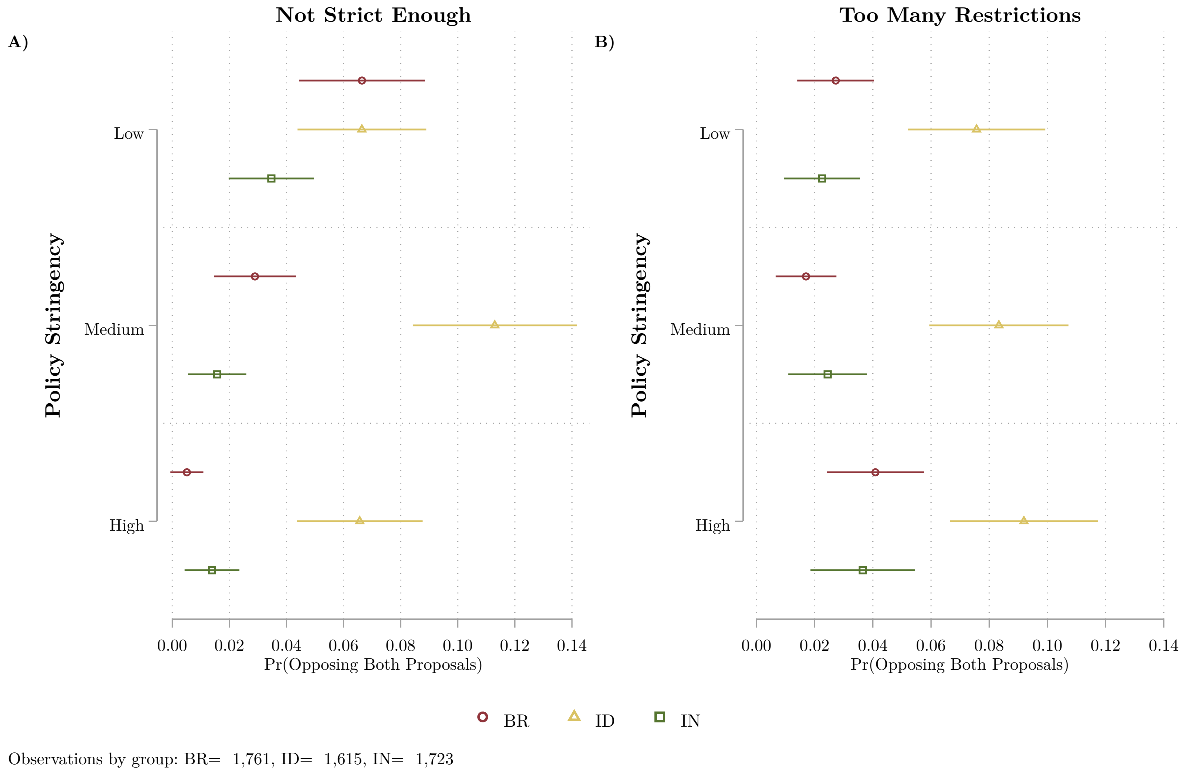

Figure S5: **Predicted probabilities for reasons of opposition (i.e. 'no to A' AND 'no to B') to supply chain policy packages in BR, ID, and IN (with 95% confidence intervals).**

References

[1] Grossman, G. & Kruger, A. Economic Growth and the Environment. *Quarterly Journal of Economics* **110**, 353–377, DOI: 10.2307/2118443 (1995).

[2] Rudolph, L., Kolcava, D. & Bernauer, T. Public demand for extraterritorial environmental and social public goods provision. *British Journal of Political Science* **https://www.doi.org/10.1017/S0007123422000175**, DOI: 10.1017/S0007123422000175 (2022).

[3] Dasgupta, S., Laplante, B., Wang, H. & Wheeler, D. Confronting the environmental Kuznets curve. *Journal of economic perspectives* **131**, 415–25, DOI: 10.1016/j.jenvman.2013.10.002 (2002). 1011.1669v3.

[4] Stern, D. I. The Rise and Fall of the Environmental Kuznets Curve. *World Development* **32**, 1419–1439, DOI: 10.1016/j.worlddev.2004.03.004 (2004).

[5] Roca, J. Do individual preferences explain the Environmental Kuznets curve? *Ecological Economics* **45**, 3–10, DOI: 10.1016/S0921-8009(02)00263-X (2003).

[6] Martínez-Alier, J. The environment as a luxury good or "too poor to be green"? *Ecological Economics* **13**, 1–10, DOI: 10.1016/0921-8009(94)00062-Z (1995).

[7] Inglehart, R. Features public support for environmental protection: Objective problems and subjective values in 43 societies. *Political Science and Politics* **28**, 57–72, DOI: 10.2307/420583 (1995). gr-qc/9809069v1.

[8] Cole, M. Trade, the Pollution Haven Hypothesis and Environmental Kuznets Curve: Examining the Linkages. *Ecological Economics* **48**, 71–81, DOI: 10.1016/j.ecolecon.2003.09.007 (2004).

[9] Spilker, G., Koubi, V. & Bernauer, T. International Political Economy and the Environment. In *Oxford Research Encyclopedia of Politics*, DOI: 10.1093/acrefore9780190228637.013.172 (Oxford University Press, 2017).

[10] Mayda, A. M. & Rodrik, D. Why are some people (and countries) more protectionist than others? *European Economic Review* **49**, 1393–1430, DOI: 10.1016/j.euroecorev.2004.01.002 (2005).

[11] Mansfield, E. D. & Mutz, D. C. Support for Free Trade. *International Organization* **63**, 425–457, DOI: 10.1017/S002U8183lMMM (2009).

[12] Kolcava, D., Smith, E. K. & Bernauer, T. Cross-national public acceptance of sustainable global supply chain policy instruments. *Nature Sustainability* **6**, 69–80, DOI: 10.1038/s41893-022-00984-8 (2022).

[13] Steg, L., Dreijerink, L. & Abrahamse, W. Why are energy policies acceptable and effective? *Environment and behavior* **38**, 92–111 (2006).

[14] Stadelmann-Steffen, I. & Dermont, C. The unpopularity of incentive-based instruments: What improves the cost–benefit ratio? *Public Choice* **175**, 37–62, DOI: 10.1007/s11127-018-0513-9 (2018).

[15] Schmalensee, R. & Stavins, R. N. The design of environmental markets: What have we learned from experience with cap and trade? *Oxford Review of Economic Policy* **33**, 572–588 (2017).

[16] Metcalf, G. E. & Weisbach, D. The design of a carbon tax. *Harv. Envtl. L. Rev.* **33**, 499 (2009).

[17] Wicki, M., Huber, R. A. & Bernauer, T. Can policy-packaging increase public support for costly policies? Insights from a choice experiment on policies against vehicle emissions. *Journal of Public Policy* **40**, 599–625, DOI: 10.1017/S0143814X19000205 (2019).

[18] de Soysa, I. & Neumayer, E. False Prophet, or Genuine Savior? Assessing the Effects of Economic Openness on Sustainable Development, 1980–99. *International Organization* **59**, 731–772, DOI: 10.1017/S0020818305050253 (2005).

[19] Vogel, M. P. *Environmental Kuznets Curves: A Study on the Economic Theory and Political Economy of Environmental Quality Improvements in the Course of Economic Growth* (Springer-Verlag, Berlin, 1999).

[20] Nguyen, Q. & Malesky, E. Fish or steel? New evidence on the environment-economy trade-off in developing Vietnam. *World Development* **147**, 105603, DOI: 10.1016/j.worlddev.2021.105603 (2021).

[21] Bernauer, T. & Nguyen, Q. Free Trade and/or Environmental Protection? *Global Environmental Politics* **15**, 105–129, DOI: 10.1162/GLEP_a_00327 (2015).

[22] Summers, N. & VanHeuvelen, T. Heterogeneity in the relationship between country-level affluence and environmental concern. *Social Forces* **96**, 329–360 (2017).

[23] Mayer, A. & Smith, E. K. Rethinking economic conditions and environmental attitudes: Macroeconomic effects, individual experiences, and subjectivity. *Social Currents* **4**, 342–359 (2017).

[24] Franzen, A. & Vogl, D. Two decades of measuring environmental attitudes: A comparative analysis of 33 countries. *Global Environmental Change* **23**, 1001–1008, DOI: 10.1016/j.gloenvcha.2013.03.009 (2013).

[25] Dunlap, R. E. & York, R. The globalization of environmental concern and the limits of the postmaterialist values explanation: Evidence from four multinational surveys. *The Sociological Quarterly* **49**, 529–563, DOI: 10.1111/j.1533-8525.2008.00127.x (2008).
