# Supplementary material for: Stringent sustainability regulations for global supply chains are supported across middle-income democracies
Source: Nat Commun. 2024 Feb 5;15:1049. doi: 10.1038/s41467-024-45399-5 (PMC10844325; doi:10.1038/s41467-024-45399-5)
Supplement: Supplementary file 3 — Reporting Summary [file 41467_2024_45399_MOESM3_ESM.pdf]

nature portfolio

Corresponding author(s): E. Keith Smith

Last updated by author(s): Dec 11, 2023

Reporting Summary

Nature Portfolio wishes to improve the reproducibility of the work that we publish. This form provides structure for consistency and transparency in reporting. For further information on Nature Portfolio policies, see our Editorial Policies and the Editorial Policy Checklist.

Statistics

For all statistical analyses, confirm that the following items are present in the figure legend, table legend, main text, or Methods section.

n/a	Confirmed	
☐	☒	The exact sample size (n) for each experimental group/condition, given as a discrete number and unit of measurement
☐	☒	A statement on whether measurements were taken from distinct samples or whether the same sample was measured repeatedly
☒	☐	The statistical test(s) used AND whether they are one- or two-sided<br>*Only common tests should be described solely by name; describe more complex techniques in the Methods section.*
☐	☒	A description of all covariates tested
☒	☐	A description of any assumptions or corrections, such as tests of normality and adjustment for multiple comparisons
☐	☒	A full description of the statistical parameters including central tendency (e.g. means) or other basic estimates (e.g. regression coefficient) AND variation (e.g. standard deviation) or associated estimates of uncertainty (e.g. confidence intervals)
☒	☐	For null hypothesis testing, the test statistic (e.g. F, t, r) with confidence intervals, effect sizes, degrees of freedom and P value noted<br>*Give P values as exact values whenever suitable.*
☒	☐	For Bayesian analysis, information on the choice of priors and Markov chain Monte Carlo settings
☒	☐	For hierarchical and complex designs, identification of the appropriate level for tests and full reporting of outcomes
☒	☐	Estimates of effect sizes (e.g. Cohen's d, Pearson's r), indicating how they were calculated

Our web collection on statistics for biologists contains articles on many of the points above.

Software and code

Policy information about availability of computer code

Data collection: No custom code was used to collect data

Data analysis: Stata replication code for analyses is available at the Open Science Foundation with the identifier doi.org/10.17605/OSF.IO/YXNW8.

For manuscripts utilizing custom algorithms or software that are central to the research but not yet described in published literature, software must be made available to editors and reviewers. We strongly encourage code deposition in a community repository (e.g. GitHub). See the Nature Portfolio guidelines for submitting code & software for further information.

Data

Policy information about availability of data

All manuscripts must include a data availability statement. This statement should provide the following information, where applicable:

- Accession codes, unique identifiers, or web links for publicly available datasets
- A description of any restrictions on data availability
- For clinical datasets or third party data, please ensure that the statement adheres to our policy

The survey instrument, replication data and coding are available at Open Science Framework (OSF) with the identifier doi.org/10.17605/OSF.IO/YXNW8.

Research involving human participants, their data, or biological material

Policy information about studies with human participants or human data. See also policy information about sex, gender (identity/presentation), and sexual orientation and race, ethnicity and racism.

Reporting on sex and gender	Gender identity (male or female) was utilized as a quota for this survey data collection to ensure representation by demographic groups within each country. Gender was interlocked with age (e.g. females 18-34) for these quotas
Reporting on race, ethnicity, or other socially relevant groupings	We also included quotas for educational attainment, with country specific (low, medium and high) categories
Population characteristics	Voting-age (18+) citizens within each country.
Recruitment	We sampled from voting-age (18) citizens within Dynata's online panel, adopting representative quotas on age, gender (interlocked with age), and education (3 categories). Respondents were compensated for their participation in accordance with their agreement with Dynata (typically in rewards and travel points). As we sampled using non-proportional methods, there is potential for self-selection biases for the respondents that participated in the study. As such, generalizability of the results to full populations should be taken with caution.
Ethics oversight	The recruitment, data collection and storage, and survey instrument were approved by the ETH-Zurich Ethics Commission (EK-2021-N67). Informed consent was obtained from respondents before their participation in the study, and respondents can end their participation and revoke their data at any time. The study design was pre-registered on the Open Science Framework (see: https://doi.org/10.17605/OSF.IO/7ATUP)

Note that full information on the approval of the study protocol must also be provided in the manuscript.

Field-specific reporting

Please select the one below that is the best fit for your research. If you are not sure, read the appropriate sections before making your selection.

☐ Life sciences ☒ Behavioural & social sciences ☐ Ecological, evolutionary & environmental sciences

For a reference copy of the document with all sections, see nature.com/documents/nr-reporting-summary-flat.pdf

Behavioural & social sciences study design

All studies must disclose on these points even when the disclosure is negative.

Study description	The study utilizes quantitative methodologies (citizen-level surveys) to identify support for supply chain regulations. We use survey embedded choice experiments (conjoint analyses), which are common to political science research. We analyze the results of these experimental designs using regression based estimates.
Research sample	We sample n=2,000 residents of the following 3 developing countries, all of a population aged 18+ (Brazil, Indonesia, India) and 12 OECD countries (Belgium, Canada, Germany, France, Italy, Spain, Netherlands, South Korea, Japan, Swizerland, United States, United Kingdom). Three developing countries were chosen as they have large volumes of exports to OECD countries (the three largest democratic, non-OECD countries), and would be therefore more strongly affected by supply chain regulations. We also chose Brazil, India and Indonesia, as they vary in terms of their industrialization, economic development, exports, and exporting partners. The OECD countries included in this study represent the 12 largest importing economies within the OECD.
Sampling strategy	We utilized Dynata, a commercial panel provider, to provide access to respondents. Respondents were sampled from the Dynata panel in each of these countries using quotas for age*gender and education. The sampling strategy utilised a non-proportional sampling design, where respondents were recruited by Dynata and could 'opt-in' to participate in the study. As the study is exploratory, and does not have many previously existing findings to rely upon for calibration, expected effect sizes were not available - limiting the ability for pre- data collection power analyses. Accordingly, sample sizes were chosen to maximize the potential number of respondents in each country given the research budget available (n=2,000 for each country). Respondent completed 5 rounds of the conjoint survey, developing the equivalent of 10,000 responses for each country - a substantial sample size which would allow for identification of significant effects of comparatively smaller sizes.
Data collection	Data was collected using an online survey platform, Qualtrics. Respondents were sent an anonymized link to the Qualtrics survey. The survey data responses are held exclusively by the research team, only meta- and aggregated-data (e.g. average completion time, quotas) were shared with Dynata. As the data was collected online, there was no researcher present during data collection. The data is confidential, and no specific identifiable information was collected from respondents. All experimental treatments were randomly assigned, and blinded to the participant.
Timing	Data was collected from April 27 and May 23, 2022 for the developing states, and from September 22 and November 3, 2021 for the OECD countries.
Data exclusions	Respondents were excluded for the following reasons; (i) they did not provide consent to participate, (ii) they were excluded as a demographic quota was already filled, (iii) they did not complete the survey, (iv) they failed at least 2 of 3 data quality checks (a)

	response duration below 45% of the median duration b) incorrect response to how many wheels a bicycle has c) and incorrect answer on an item requesting respondents to `select the triangle'.
Non-participation	Respondents which failed at least two of the three attention check items were replaced in the sampling process, and therefore excluded from empirical analysis (3.2%)
Randomization	Respondents were randomized into two different experimental designs. First, they were presented with conjoint experiments that present randomly assigned levels for each attribute of the policy design. Second, they were assigned to 'low', 'medium', or 'high' policy vignettes, which they are tasked with evaluating for the "probes".

Reporting for specific materials, systems and methods

We require information from authors about some types of materials, experimental systems and methods used in many studies. Here, indicate whether each material, system or method listed is relevant to your study. If you are not sure if a list item applies to your research, read the appropriate section before selecting a response.

Materials & experimental systems

n/a	Involved in the study
☒	☐ Antibodies
☒	☐ Eukaryotic cell lines
☒	☐ Palaeontology and archaeology
☒	☐ Animals and other organisms
☒	☐ Clinical data
☒	☐ Dual use research of concern
☒	☐ Plants

Methods

n/a	Involved in the study
☒	☐ ChIP-seq
☒	☐ Flow cytometry
☒	☐ MRI-based neuroimaging
